# Supplementary figures and images for: Applications of Nano/Micromotors for Treatment and Diagnosis in Biological Lumens
Source: Micromachines (Basel). 2022 Oct 19;13(10):1780. doi: 10.3390/mi13101780 (PMC9610721; doi:10.3390/mi13101780)

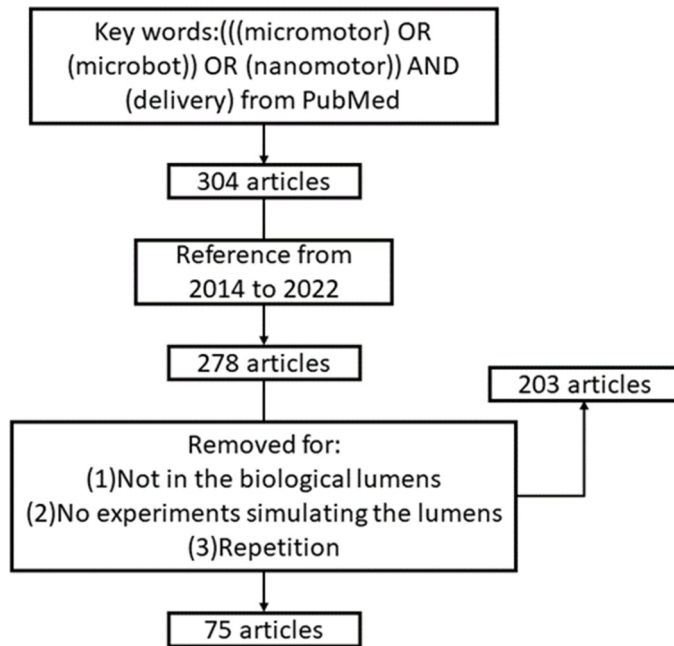

**Figure S1:** Review strategy.

Supplement: Supplementary file 1 [file micromachines-13-01780-s001.zip › micromachines-1926434-supplementary.pdf]
